# Aquaporins Transcripts with Potential Prognostic Value in Pancreatic Cancer

**DOI:** 10.3390/genes14091694

**Published:** 2023-08-25

**Authors:** Paula A. Lopes, Elisabete Fonseca, Inês V. da Silva, Emanuel Vigia, Jorge Paulino, Graça Soveral

**Affiliations:** 1 CIISA—Centro de Investigação Interdisciplinar em Sanidade Animal, Faculdade de Medicina Veterinária, Universidade de Lisboa, 1300-477 Lisbon, Portugal; 2 Laboratório Associado para Ciência Animal e Veterinária (AL4AnimalS), Faculdade de Medicina Veterinária, Universidade de Lisboa, 1300-477 Lisbon, Portugal; 3 Research Institute for Medicines (iMed.ULisboa), Faculdade de Farmácia, Universidade de Lisboa, 1649-003 Lisbon, Portugal; es.fonseca@campus.fct.unl.pt (E.F.); imsilva1@campus.ul.pt (I.V.d.S.); 4 Department of Pharmaceutical Sciences and Medicines, Faculdade de Farmácia, Universidade de Lisboa, 1649-003 Lisbon, Portugal; 5 Hepatobiliopancreatic and Transplantation Center, Hospital de Curry Cabral-CHULC, 1050-099 Lisbon, Portugal; vigiacarla@gmail.com; 6 NOVA Medical School, Universidade NOVA de Lisboa, 1169-056 Lisbon, Portugal; fusilis@gmail.com; 7 Hospital da Luz, 1500-650 Lisbon, Portugal

**Keywords:** aquaporins, transcriptional profile, pancreas, pancreatic cancer

## Abstract

Pancreatic cancer is anticipated to be the second leading cause of cancer-related death by 2030. Aquaporins (AQPs), a family of water channel proteins, have been linked to carcinogenesis. The aim of this study was to determine AQP gene expression in pancreatic cancer tissues and to validate aquaporins as possible diagnosis and/or prognosis genes. The relative gene expression levels of AQP1, AQP3, AQP5, and AQP9 were analyzed using real-time quantitative PCR (RT-qPCR) in 24 paired pancreatic tumors and adjacent healthy tissues according to variables such as age, gender, and tumor invasiveness and aggressiveness. AQPs transcripts were detected in both healthy and tumor tissues. While AQP1 was downregulated in the tumor samples, AQP3 was particularly overexpressed in low-grade invasive tumors. Interestingly, most of the strong positive Pearson correlation coefficients found between AQPs in healthy tissues were lost when analyzing the tumor tissues, suggesting disruption of the coordinated AQP-gene expression in pancreatic cancer.

## 1. Introduction

Pancreatic cancer is the 7th most lethal and 14th most prevalent type of cancer with a five-year survival rate of only 2–9%. Both incidence and mortality are higher in males than in females [1]. About 85% of pancreatic cancers are ductal adenocarcinomas (PDACs), which arise in the exocrine section of the gland and represent one of the most aggressive human malignancies. The three well-characterized human PDAC precursor lesions, intraepithelial neoplasms (PanINs), mucinous cystic neoplasms (MCNs), and intraductal papillary mucinous neoplasms (IPMNs), may all develop into a malignant PDAC [2]. Late diagnosis based on vague symptoms is a contributing factor to the low survival rate, which results in unresectable tumors in 80–85% of cases [3]. The incidence and death rates of pancreatic cancer have continued to increase, predictively rendering this disease the second leading cause of cancer-related death by 2030 [4]. The only therapeutic option for treating PDAC is surgery, and due to the lack of distinguishing symptoms and clear clinical signs, an early PDAC diagnosis is still challenging.

The patient’s age, gender, ethnicity, and genetic background are among several non-modifiable risk factors associated with pancreatic cancer [5], which is more frequent in elderly individuals and extremely rare before the age of 30. According to the Global Cancer Observatory (GLOBOCAN), in 2020, pancreatic cancer was the 13th most common cause of cancer in males and the 12th in females [1,6]. The only clinically approved biomarker for pancreatic cancer is carbohydrate antigen 19-9 (CA 19-9) with a sensitivity range of 70–92% and a specificity of 68–92% [7,8]. However, the high number of false positive and false negative results, together with the lack of sensitivity for early detection, limits CA 19-9’s accuracy and usefulness [9].

Aquaporins (AQPs) are a family of water channel proteins that facilitate the permeation of water (orthodox aquaporins) and other small non-charged solutes, such as glycerol, across membranes (aquaglyceroporins) [10]. The 13 AQPs found in humans (AQP0–AQP12) have been detected not only in tissues involved in fluid transport but also in non-absorbing or non-excretory tissues [11]. In addition to acting as membrane channels, AQPs have multiple functions beyond their canonic role; they facilitate cell morphology changes [12], modulate membranes’ biomechanical properties [13], and are involved in signaling pathways controlling cell migration and cell–cell adhesion [14,15,16,17], therefore impacting the initiation and development of carcinogenesis.

In the last decade, abnormal expression levels of AQPs have been reported in multiple cancer types [10,14,18] and specifically associated with the mechanisms of migration and proliferation. In the pancreas, AQPs are expressed in the endo- and exocrine pancreas, with them being involved in insulin and pancreatic fluid secretion, respectively [19,20,21,22]. Several pancreatic conditions, including cancer, have been associated with altered AQP expression [23]. In human pancreatic cancer, aberrant AQP expression was found to be associated with tumor pathological grade, clinical stage, impaired overall survival [24], and even resistance to chemotherapy [23,25]. Strong evidence highlights the value of AQPs as biomarkers for cancer diagnosis and prognosis. Using transcriptome data mining techniques on three independent PDAC datasets, a recent study reported the differential expression of a panel of genes, including the expression profile of the AQP interactome. Among the 20 differentially expressed genes identified (downregulated and upregulated), a molecular panel of four genes was identified as potential prognostic markers associated with overall survival, highlighting the urgent need for in vitro testing to evaluate their potential prognostic role and validate their clinical applicability [26].

In this study, we analyzed the gene expression levels of AQPs in paired pancreatic cancer tissues of different histological types and adjacent healthy tissues removed from patients during curative surgical resection. We present a descriptive study in which AQP expression levels were analyzed according to various factors, such as the patient’s age, gender, and tumor invasiveness and aggressiveness, aiming to evaluate their potential prognosis usefulness in pancreatic cancer.

## 2. Materials and Methods

### 2.1. Ethical Approval and Sample Characterization

All procedures involving human participants were performed in line with the ethical standards of the institutional and national research committee and the 1964 Helsinki Declaration and its later amendments or comparable ethical standards [27]. Tumor samples and matched adjacent non-neoplastic samples were obtained from 24 patients who underwent surgery. Tissue was obtained and used in a manner compliant with the Declaration of Helsinki, as revised in 1983. This study was approved by the ethics committee of the Centro Hospitalar of Lisboa Central CHULC (INV-106, 2021). All patients were classified according to the pathological tumor/node/metastasis (pTNM) system and based on the 7th edition of the American Joint Committee on Cancer (AJCC).

The cohort’s clinico-pathological features are described in Table 1. The samples were collected from 12 male and 12 female patients, with ages ranging from 51 to 82 years at the time of surgery and were classified according to the aggressiveness and invasiveness grade of pancreatic cancer by the surgeons and pathologists who provided the sample stratification. Aggressiveness was defined according to the tumor histological type and grade and was classified from 1 to 4. The samples were grouped into a low-grade aggressive group (patients with aggressiveness ≤ 2) and a high-grade aggressive group (patients with aggressiveness ≥ 3). The invasiveness grade was defined according to tumor local invasion and lymph node status and was classified from 1 to 4. The samples were grouped into a low-grade invasive group (patients with an invasiveness grade = 1) and a high-grade invasive group (patients with an invasiveness grade ≥ 2).

### 2.2. RNA Isolation and RT-qPCR Analysis

For gene expression analysis, healthy and tumor tissues were homogenized in TRIzol, and RNA was extracted according to the manufacturer’s protocol (Ambion, Waltham, MA, USA). After the mRNA concentration and quality were evaluated, reverse transcription was performed using an NZY First-Strand cDNA Synthesis Kit according to the manufacturer’s protocol (NZYTech, Lisbon, Portugal), as previously described [28]. AQP1, AQP3, AQP5, AQP9, and β-actin (housekeeping gene) expression was assessed by quantitative PCR using TaqMan™ Universal PCR Master Mix II with UNG and TaqMan™ assays (Table 2) (Applied Biosystems, Waltham, MA, USA).

Analysis of the relative gene expression data was performed using real-time quantitative PCR (RT-qPCR) and calculated according to the following formula: relative gene expression = 2^CT housekeeping^/2^CT target^. To calculate the gene expression level, the Ct values were normalized to the mean of the housekeeping gene, ACTB (β-actin). The relative expression levels were then calculated as a variation of the Livak method [29], corrected for variation in the amplification efficiency, as previously described [30].

### 2.3. Statistical Analysis

Statistical analysis was carried out using the generalized linear mixed (GLM) model of Statistical Analysis System (SAS) software, version 9.4 [31]. Once normality was tested by the Kolmogorov–Smirnov test and variance was tested homogeneity by Levene’s test, significant multiple comparisons were carried out using the PDIFF option adjusted with Tukey–Kramer to determine any statistical differences among the healthy and tumor tissues. Pearson’s correlation coefficients were calculated using the Proc CORR procedure of SAS to establish linear relationships among the genes’ expression. Data were presented as the mean and standard error of the mean (SEM). A *p*-value lower than 0.05 was taken as being statistically significant.

## 3. Results and Discussion

**Figure 1:**
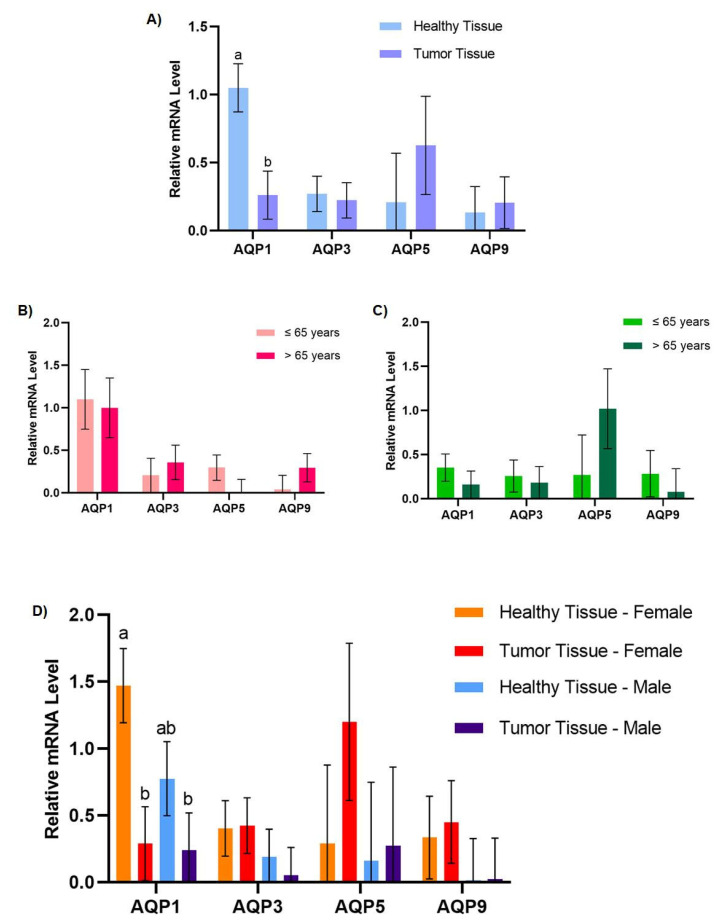
Effects of age range and gender on the relative gene expression levels of AQP1, AQP3, AQP5, and AQP9 in healthy and tumor tissues. The values are means, with their standard errors represented by vertical bars. ^a,b^ Mean values with unlike letters were significantly different (Tukey’s post hoc, *p* < 0.05). (**A**) Effect of tissue type, healthy (*n* = 24) and tumor (*n* = 24) tissues, on the relative gene expression levels of AQP1, AQP3, AQP5, and AQP9. (**B**) Effect of age range, ≤65 years (*n* = 14) and >65 years (*n* = 10), on the relative gene expression levels of AQP1, AQP3, AQP5, and AQP9 in healthy tissues (*n* = 24). (**C**) Effect of age range, ≤65 years (*n* = 14) and >65 years (*n* = 10), on the relative gene expression levels of AQP1, AQP3, AQP5, and AQP9 in tumor tissues (*n* = 24). (**D**) Effect of tissue type, healthy (*n* = 24) and tumor (*n* = 24), and gender, males (*n* = 12) and females (*n* = 12), on the relative gene expression levels of AQP1, AQP3, AQP5, and AQP9 (normalized to ACTB).

AQP1, AQP3, AQP5, and AQP9 were detected in both healthy and tumor tissues (Figure 1A). While for AQP3, AQP5, and AQP9 no statistically significant differences were found between the healthy and tumor samples (*p* > 0.05), the AQP1 mRNA expression levels were downregulated in tumor tissues (*p* = 0.003). Interestingly, in a previous study, AQP1 was found to be overexpressed in PDAC [32]; however, probably due to the diverse pancreatic cancer histological types included in the present study, this reported upregulation could not be reproduced herein for the AQP1 transcripts. In our previous study, which analyzed human biopsies of PDAC using histochemistry, both AQP3 and AQP5 protein expression were found to be upregulated in moderately differentiated tumors [24]. Here, we also observed a tendency for AQP5 gene upregulation in tumor tissues, and interestingly, upregulation of AQP5 at the protein level, which correlated with aspects of cancer biology, was also observed in previous studies [24]. In fact, the role of AQP5 in facilitating hydrogen peroxide membrane permeation and contributing to cellular oxidative stress was associated with pancreatic cancer cell migration, promoting invasiveness [33].

To investigate a possible correlation between AQP gene expression and the incidence of pancreatic cancer by age and gender, the paired tumor and healthy adjacent samples were then divided according to the patients’ age range (Figure 1B,C) and gender (female and male) (Figure 1D). No significant differences were found when dividing the data by age (*p* > 0.05). Data about gender dependency in AQP expression are scarce; however, adipose AQP7 is more abundant in females than in males [34]. Additionally, evidence suggests that hepatic AQP9 might also present sexual dimorphism [34]. In this study, AQP1 expression was found to be downregulated in the tumor tissues of both genders (Figure 1D). Although the AQP1 expression levels were not different between healthy tissues from males and females (*p* > 0.05) nor between tumor tissues from males and females (*p* > 0.05), in female patients, a significant difference (*p* = 0.007) was found between healthy and tumor tissues, where AQP1 was markedly downregulated. As for AQP3, AQP5, and AQP9, the relative steady-state levels were not found to be significantly different for any of the variables mentioned above (*p* > 0.05) (Figure 1D).

**Figure 2:**
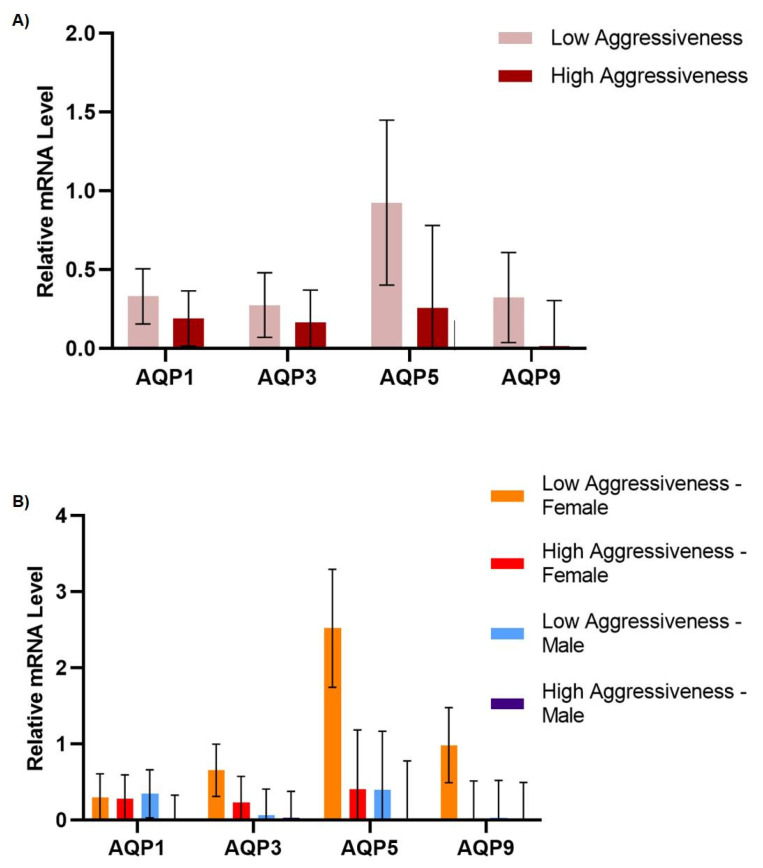
Effects of gender and aggressiveness grade on the relative gene expression levels of AQP1, AQP3, AQP5, and AQP9. The values are means, with their standard errors represented by vertical bars. (**A**) Effect of aggressiveness grade, low (*n* = 14) and high (*n* = 10), on the relative gene expression levels of AQP1, AQP3, AQP5, and AQP9. (**B**) Effect of aggressiveness grade, low (*n* = 14) and high (*n* = 10), and gender, male (*n* = 12) and female (*n* = 12), on the relative gene expression levels of AQP1, AQP3, AQP5, and AQP9 (normalized to ACTB).

Regarding tumor aggressiveness, AQP gene expression levels were not found to be different between low- and high-grade aggressive tumors (Figure 2A) (*p* > 0.05) nor did they vary significantly (*p* > 0.05) according to gender or the aggressiveness grade (Figure 2B).

**Figure 3:**
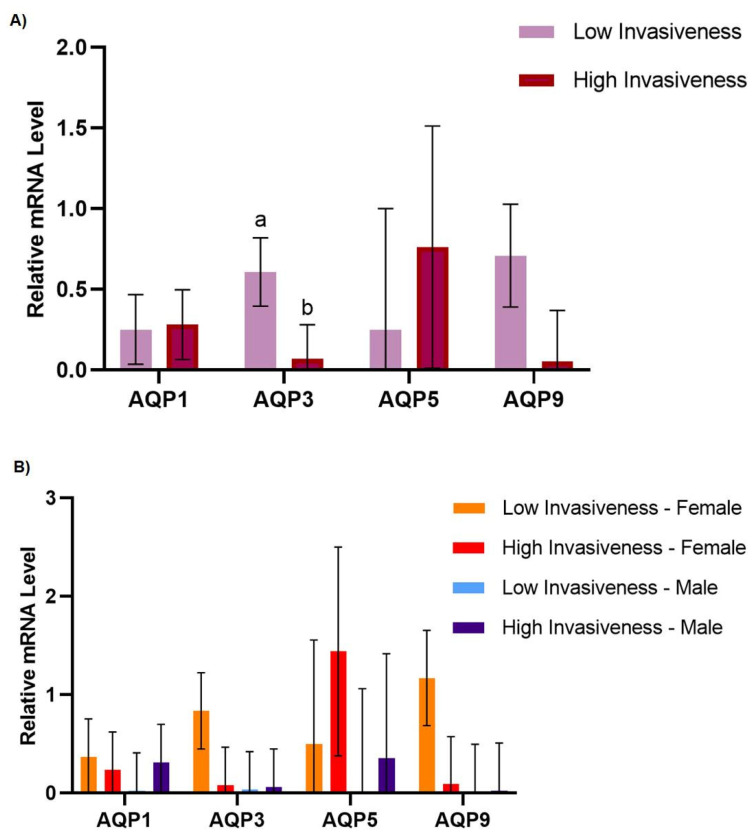
Effect of gender and invasiveness grade on the relative gene expression levels of AQP1, AQP3, AQP5, and AQP9. The values are means, with their standard errors represented by vertical bars. ^a,b^ Mean values with unlike letters were significantly different (Tukey’s post hoc, *p* < 0.05). (**A**) Effect of invasiveness grade, low (*n* = 7) and high (*n* = 17), on the relative gene expression levels of AQP1, AQP3, AQP5, and AQP9. (**B**) Effect of invasiveness grade, low (*n* = 7) and high (*n* = 17), and gender, male (*n* = 12) and female (*n* = 12), on the relative gene expression levels of AQP1, AQP3, AQP5, and AQP9 (normalized to ACTB).

Regarding tumor invasiveness, while AQP1 mRNA levels were identical in low- and high-grade invasive tumors (Figure 3A) (*p* > 0.05), AQP3 levels were higher in low-grade invasive tumors than in high-grade invasive tumors, suggesting an inverse correlation between AQP3 expression and pancreatic tumor invasiveness (*p* = 0.046) (Figure 3A). It is worth mentioning that several studies have revealed AQP3’s association with cancer aggressiveness and invasiveness in diverse types of cancer. In fact, AQP3 has been reported as a crucial player in cell migration and invasion in breast cancer, cell migration and adhesion in pancreatic cancer, and invasiveness in prostate cancer [13,35,36,37]. Interestingly, through a large-scale proteomic analysis, AQP3 was found to be significantly associated with the activity of mTOR signaling [38]. This study suggested that AQP3 can promote the tumor growth of pancreatic cancer cells by activating the mTOR signaling pathway, shedding some light on the molecular mechanisms that are potentially involved.

The AQP9 gene expression levels did not vary between low- and high-grade invasive tumors (*p* = 0.090) (Figure 3A). AQP5 displayed different dynamics (Figure 3A), but no differences were observed regarding invasiveness (*p* = 0.550).

Although all the tested AQPs displayed different levels of gene expression depending on invasiveness and gender (Figure 3B), none of these results were statistically significant (*p* > 0.05). Overall, the AQP expression levels were not determined by age or gender, even when considering the aggressiveness and invasiveness stages.

Pearson’s correlation coefficient, also called the correlation coefficient, is a mathematical measurement that is used to quantify the strength of the association between two variables. Pearson’s correlation coefficient, r, takes on the values of −1 through +1. Values of −1 or +1 indicate a perfect linear relationship between the two variables, whereas a value of 0 indicates no linear relationship. Negative values simply indicate the direction of the association, whereby as one variable increases, the other decreases [31].

Interestingly, the Pearson’s correlation coefficients between the AQP transcripts in healthy and tumor tissues were found to be positive, revealing either high or moderate correlations (high correlation, r > 0.7; moderate correlation, 0.7 ≥ r ≥ 0.3; low correlation, r < 0.3) according to Costa et al. [39].

**Figure 4:**
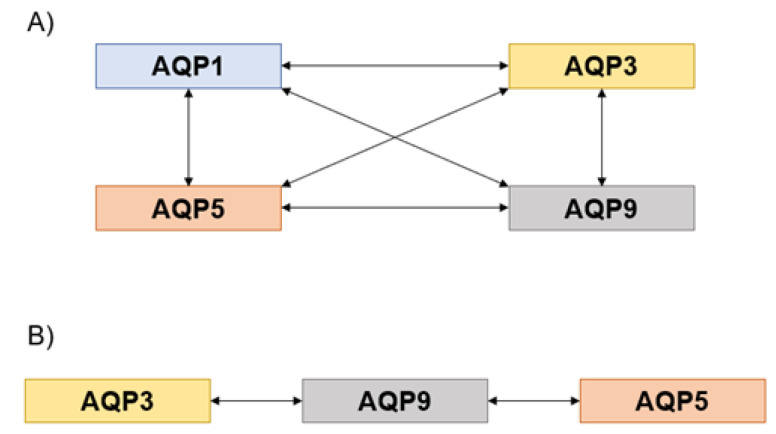
Pearson’s correlations. (**A**) Diagram of the Pearson’s correlations between AQPs in healthy tissues. (**B**) Diagram of the Pearson’s correlations among AQPs in pancreatic tumor tissues.

In healthy tissues, AQP3 was found to be correlated with AQP1 (r = 0.701, *p* = 0.004); AQP5 was correlated with AQP1 (r = 0.902, *p* = 0.001) and AQP3 (r = 0.647, *p* = 0.031); and AQP9 was correlated with AQP1 (r = 0.859, *p* = 0.007), AQP3 (r = 0.986, *p* < 0.001), and AQP5 (r = 0.915, *p* = 0.002) (Figure 4A). However, in tumor tissues, AQP9 only correlated with AQP3 (r = 0.992, *p* < 0.001) and AQP5 (r = 0.975, *p* < 0.001). No correlations were found between AQP1 and the other AQPs (*p* > 0.05) (Figure 4B). AQP1 was not correlated with AQP3 (r = 0.170, *p* = 0.438), AQP5 (r = 0.073, *p* = 0.752), nor AQP9 (r = 0.132, *p* = 0.581). Therefore, according to the Pearson’s analysis, all AQPs correlated with each other in healthy tissues. Interestingly, while AQP1 and AQP5 are orthodox water channels, AQP3 and AQP9 are aquaglyceroporins, and all these isoforms also permeate hydrogen peroxide [40], suggesting that the different features of each AQP are important to ensure the pancreas’ physiological function [20]. In the tumor tissues, most of the above-mentioned correlations disappeared, suggesting that the regulation coordinated by gene expression observed in healthy tissues is disrupted by the initiation of pancreatic cancer. In fact, in the tumor samples, AQP3 correlated with AQP9 while AQP9 also correlated with AQP5. AQP1 lost correlations with any other AQP. Overall, these data suggest that once cells undergo carcinogenesis, AQP gene expression is deregulated, impacting on how each isoform influences the others.

In conclusion, this study revealed novel findings on the role of AQPs in pancreatic cancer, most notably the downregulation of AQP1 in tumor tissues and the increased expression of AQP3 in less-invasive tumors. These results pave the way for future research into the diagnostic and prognostic value of AQPs in pancreatic cancer. However, it is important to note that the results of this study are preliminary and should be interpreted with caution due to the small sample size and the varied histological types of pancreatic cancer involved. Further research involving a larger cohort of patients is needed to validate these findings and to better understand the complex role of AQPs in the development and progression of pancreatic cancer.

## Figures and Tables

**Table 1:** Clinico-pathological characterization of tumor samples.

Patient	Gender	Age	Cancer Type	Aggressiveness Grade ^1^	Invasiveness Grade ^2^
1	M	69	Ampulla adenocarcinoma	Low	High
2	M	55	Kidney metastasis	Low	Low
3	F	55	Ductal adenocarcinoma	High	High
4	M	63	Ampulla adenocarcinoma	Low	High
5	M	69	Distal cholangiocarcinoma	High	High
6	M	59	Ductal adenocarcinoma	High	High
7	F	69	Non-invasive intraductal papillary mucinous neoplasm	Low	Low
8	F	52	Invasive intraductal papillary mucinous neoplasm	Low	High
9	F	69	Endocrine neoplasia	High	Low
10	F	82	Ductal adenocarcinoma	High	High
11	M	58	Distal cholangiocarcinoma	Low	High
12	F	65	Non-invasive intraductal papillary mucinous neoplasm	Low	Low
13	F	65	Ductal adenocarcinoma	High	High
14	M	80	Ductal adenocarcinoma	Low	High
15	F	72	Ampulla adenocarcinoma	Low	High
16	F	60	Ductal adenocarcinoma	High	High
17	M	56	Ampulla adenocarcinoma	Low	High
18	M	56	Cystic neuroendocrine tumor	Low	Low
19	M	67	Ductal adenocarcinoma	High	High
20	F	78	Ductal adenocarcinoma	High	High
21	M	50	Distal cholangiocarcinoma	Low	High
22	F	80	Invasive intraductal papillary mucinous neoplasm	Low	Low
23	M	64	Ductal adenocarcinoma	High	High
24	F	51	Cystic neuroendocrine tumor	Low	Low

^1^ The aggressiveness grade was defined according to the tumor histological type and grade and was classified from 1 to 4. The samples were grouped into a low-grade aggressive group (patients with aggressiveness ≤ 2) and a high-grade aggressive group (patients with aggressiveness ≥ 3). ^2^ The invasiveness grade was defined according to the tumor local invasion and lymph node status. The samples were grouped into a low-grade invasive group (patients with an invasiveness grade = 1) and a high-grade invasive group (patients with an invasiveness grade ≥ 2).

**Table 2:** Aquaporins and corresponding housekeeping gene-specific probes used for RT-qPCR.

Gene	Full Gene Name	TaqMan Gene Expression Assay
AQP1	Aquaporin 1	Hs01028916_m1
AQP3	Aquaporin 3	Hs01105469_g1
AQP5	Aquaporin 5	Hs00387048_m1
AQP9	Aquaporin 9	Hs00175573_m1
Housekeeping Gene		
ACTB	β-actin	Hs99999903_m1

## Data Availability

The data presented in this study are available in this article. Datasets are accessible to readers from the corresponding author on request.

## References

[1] Sung H., Ferlay J., Siegel R.L., Laversanne M., Soerjomataram I., Jemal A., Bray F. (2021). Global cancer statistics 2020: GLOBOCAN estimates of incidence and mortality worldwide for 36 cancers in 185 countries. CA A Cancer J. Clin..

[2] Maitra A., Fukushima N., Takaori K., Hruban R.H. (2005). Precursors to invasive pancreatic cancer. Adv. Anat. Pathol..

[3] McGuigan A., Kelly P., Turkington R.C., Jones C., Coleman H.G., McCain R.S. (2018). Pancreatic cancer: A review of clinical diagnosis, epidemiology, treatment and outcomes. World J. Gastroenterol..

[4] Rahib L., Smith B.D., Aizenberg R., Rosenzweig A.B., Fleshman J.M., Matrisian L.M. (2014). Projecting cancer incidence and deaths to 2030: The unexpected burden of thyroid, liver, and pancreas cancers in the United States. Cancer Res..

[5] Midha S., Chawla S., Garg P.K. (2016). Modifiable and non-modifiable risk factors for pancreatic cancer: A review. Cancer Lett..

[6] Ilic M., Ilic I. (2016). Epidemiology of pancreatic cancer. World J. Gastroenterol..

[7] Ćwik G., Wallner G., Skoczylas T., Ciechański A., Zinkiewicz K. (2006). Cancer antigens 19-9 and 125 in the differential diagnosis of pancreatic mass lesions. Arch. Surg..

[8] Ballehaninna U.K., Chamberlain R.S. (2012). The clinical utility of serum CA 19-9 in the diagnosis, prognosis and management of pancreatic adenocarcinoma: An evidence based appraisal. J. Gastrointest. Oncol..

[9] Ballehaninna U.K., Chamberlain R.S. (2011). Serum CA 19-9 as a biomarker for pancreatic cancer—A comprehensive review. Indian J. Surg. Oncol..

[10] Verkman A.S. (2012). Aquaporins in clinical medicine. Annu. Rev. Med..

[11] Ishibashi K., Hara S., Kondo S. (2009). Aquaporin water channels in mammals. Clin. Exp. Nephrol..

[12] Papadopoulos M.C., Saadoun S. (2015). Key roles of aquaporins in tumor biology. Biochim. Biophys. Acta.

[13] Silva P.M., da Silva I.V., Sarmento M.J., Silva I.C., Carvalho F.A., Soveral G., Santos N.C. (2022). Aquaporin-3 and aquaporin-5 facilitate migration and cell-cell adhesion in pancreatic cancer by modulating cell biomechanical properties. Cells.

[14] Direito I., Madeira A., Brito M.A., Soveral G. (2016). Aquaporin-5: From structure to function and dysfunction in cancer. Cell. Mol. Life Sci. CMLS.

[15] Marlar S., Jensen H.H., Login F.H., Nejsum L.N. (2017). Aquaporin-3 in cancer. Int. J. Mol. Sci..

[16] Edamana S., Login F.H., Yamada S., Kwon T.H., Nejsum L.N. (2021). Aquaporin water channels as regulators of cell-cell adhesion proteins. Am. J. Physiol. Cell Physiol..

[17] Moon C.S., Moon D., Kang S.K. (2022). Aquaporins in cancer biology. Front. Oncol..

[18] Pimpão C., Wragg D., da Silva I.V., Casini A., Soveral G. (2022). Aquaglyceroporin modulators as emergent pharmacological molecules for human diseases. Front. Mol. Biosci..

[19] Delporte C. (2014). Aquaporins in salivary glands and pancreas. Biochim. Biophys. Acta.

[20] Méndez-Giménez L., Ezquerro S., da Silva I.V., Soveral G., Frühbeck G., Rodríguez A. (2018). Pancreatic aquaporin-7: A novel target for anti-diabetic drugs?. Front. Chem..

[21] da Silva I.V., Cardoso C., Méndez-Giménez L., Camoes S.P., Frühbeck G., Rodríguez A., Miranda J.P., Soveral G. (2020). Aquaporin-7 and aquaporin-12 modulate the inflammatory phenotype of endocrine pancreatic beta-cells. Arch. Biochem. Biophys..

[22] Bruun-Sørensen A.S., Edamana S., Login F.H., Borgquist S., Nejsum L.N. (2021). Aquaporins in pancreatic ductal adenocarcinoma. APMIS.

[23] Arsenijevic T., Perret J., Van Laethem J., Delporte C. (2019). Aquaporins involvement in pancreas physiology and in pancreatic diseases. Int. J. Mol. Sci..

[24] Direito I., Paulino J., Vigia E., Brito M.A., Soveral G. (2017). Differential expression of aquaporin-3 and aquaporin-5 in pancreatic ductal adenocarcinoma. J. Surg. Oncol..

[25] Edamana S., Pedersen S.F., Nejsum L.N. (2023). Aquaporin water channels affect the response of conventional anticancer therapies of 3D grown breast cancer cells. Biochem. Biophys. Res. Commun..

[26] Magouliotis D.E., Tasiopoulou V.S., Dimas K., Sakellaridis N., Svokos K.A., Svokos A.A., Zacharoulis D. (2019). Transcriptomic analysis of the aquaporin (AQP) gene family interactome identifies a molecular panel of four prognostic markers in patients with pancreatic ductal adenocarcinoma. Pancreatology.

[27] World Medical Association (2013). World Medical Association Declaration of Helsinki: Ethical principles for medical research involving human subjects. JAMA.

[28] da Silva I.V., Soares B.P., Pimpão C., Pinto R.M.A., Costa T., Freire J.P.B., Corrent E., Chalvon-Demersay T., Prates J.A.M., Lopes P.A. (2021). Glutamine and cystine-enriched diets modulate aquaporins gene expression in the small intestine of piglets. PLoS ONE.

[29] Livak K.J., Schmittgen T.D. (2001). Analysis of relative gene expression data using real-time quantitative PCR and the 2^−ΔΔCT^ method. Methods.

[30] Fleige S., Pfaffl M.W. (2006). RNA integrity and the effect on the real-time qRT-PCR performance. Mol. Asp. Med..

[31] SAS Institute (2004). SAS/STAT User’s Guide, Version 9.1.

[32] Zou W., Yang Z., Li D., Liu Z., Zou Q., Yuan Y. (2019). AQP1 and AQP3 expression are associated with severe symptoms and poor-prognosis of the pancreatic ductal adenocarcinoma. Appl. Immunohistochem. Mol. Morphol..

[33] Rodrigues C., Pimpão C., Mósca A.F., Coxixo A.S., Lopes D., da Silva I.V., Pedersen P.A., Antunes F., Soveral G. (2019). Human aquaporin-5 facilitates hydrogen peroxide permeation affecting adaption to oxidative stress and cancer cell migration. Cancers.

[34] Rodriguez A., Marinelli R.A., Tesse A., Fruhbeck G., Calamita G. (2015). Sexual dimorphism of adipose and hepatic aquaglyceroporins in health and metabolic disorders. Front. Endocrinol..

[35] Chen J., Wang Z., Xu D., Liu Y., Gao Y. (2015). Aquaporin 3 promotes prostate cancer cell motility and invasion via extracellular signal-regulated kinase 1/2-mediated matrix metalloproteinase-3 secretion. Mol. Med. Rep..

[36] Huang Y.T., Zhou J., Shi S., Xu H.Y., Qu F., Zhang D., Chen Y.-D., Yang J., Huang H.-F., Sheng J.-Z. (2015). Identification of estrogen response element in aquaporin-3 gene that mediates estrogen-induced cell migration and invasion in estrogen receptor-positive breast cancer. Sci. Rep..

[37] Satooka H., Hara-Chikuma M. (2016). Aquaporin-3 controls breast cancer cell migration by regulating hydrogen peroxide transport and its downstream cell signaling. Mol. Cell. Biol..

[38] Huang X., Huang L., Shao M. (2017). Aquaporin 3 facilitates tumor growth in pancreatic cancer by modulating mTOR signaling. Biochem. Biophys. Res. Commun..

[39] Costa P., Lemos J.P., Lopes P.A., Alfaia C.M., Costa A.S.H., Bessa R.J.B., Prates J.A.M. (2012). Effect of low- and high-forage diets on meat quality and fatty acid composition of Alentejana and Barrosã beef breeds. Animal.

[40] da Silva I.V., Garra S., Calamita G., Soveral G. (2022). The multifaceted role of aquaporin-9 in health and its potential as a clinical biomarker. Biomolecules.

